# Primary care physicians’ perceptions of social determinants of health recommendations: a qualitative study

**DOI:** 10.3399/BJGPO.2022.0129

**Published:** 2023-01-25

**Authors:** Junki Mizumoto, Toshichika Mitsuyama, Masato Eto, Masashi Izumiya, Shoko Horita

**Affiliations:** 1 Department of Medical Education Studies, International Research Center for Medical Education, Graduate School of Medicine, The University of Tokyo, Tokyo, Japan

**Keywords:** love and breakup letter methodology, qualitative research, social determinants of health, primary health care, general practice

## Abstract

**Background:**

Several organisations have called for primary care professionals to address social determinants of health (SDoH) in clinical settings. For primary care physicians to fulfill their community health responsibilities, the implications of the SDoH recommendations need to be clarified.

**Aim:**

To describe primary care physicians’ views about being asked to address SDoH in clinical settings, from both positive and negative perspectives.

**Design & setting:**

A qualitative study in Japan. Twenty-one physicians were purposively recruited.

**Method:**

‘Love and breakup letter’ methodology was used to collect qualitative data that contained both positive and negative feelings. Participants wrote love and breakup letters about being asked to address SDoH in a clinical setting, then undertook an in-depth online interview. Data were analysed via thematic analysis using the framework approach.

**Results:**

The following themes were identified: (i) primary care physicians take pride in being expected to address SDoH; (ii) primary care physicians rely on the recommendations as a partner, even in difficult situations; (iii) primary care physicians consider the recommendations to be bothersome, with unreasonable demands and challenges, especially when supportive surroundings are lacking; and (iv) primary care physicians reconstruct the recommendations on the basis of their experience.

**Conclusion:**

Primary care physicians felt both sympathy and antipathy towards recommendations asking them to address SDoH in their clinical practice. The recommendations were not followed literally, instead contributing to physicians’ clinical mindlines. Professional organisations that plan to develop and publish recommendations about SDoH should consider how their recommendations might be perceived by their target audience.

## How this fits in

Several organisations have advocated for primary care professionals to address SDoH in a clinical setting. This study aimed to describe primary care physicians’ views regarding recommendations making this request, from both positive and negative perspectives. Participants sometimes considered the recommendations to be helpful and supportive, and sometimes as irritating and nagging, particularly when they were not in supportive surroundings. Although participants did not follow the recommendations literally, they perceived positive implications for their clinical practice.

## Introduction

SDoH are non-medical factors that influence health outcomes.^1^ It is estimated that more than half of health outcomes are determined by socioeconomic or behavioural factors.^2^ Primary care describes itself as the foundation of the healthcare system,^3^ and should play a role in addressing health inequity by recognising patients’ socioeconomic background, identifying marginalised populations, and delivering high quality preventive care and chronic disease management.^4^ Considering the reality that the distribution of primary care resources is unequal,^5–11^ addressing patients’ social conditions in primary care settings should be prioritised as an urgent issue.

Healthcare professionals’ attitudes and beliefs regarding patients contribute to inequalities in health care.^11^ Social justice is one of the moral foundations of primary care,^12^ and interventions in SDoH in a clinical setting are theoretically considered to contribute to better health outcomes, better healthcare delivery, and cost savings.^3,13^ Consequently, several organisations have called for medical professionals, including primary care professionals, to address SDoH in the medical healthcare system.^14–19^ However, primary care physicians may experience confusion in assuming responsibility for their patients’ social determinants, for several reasons.^20^ First, dealing with patients with social difficulties can be stressful and depressing.^21–23^ Second, newly detected patient social needs could lead to excessive medicalisation and impose additional work on busy professionals.^24,25^ Third, dealing with SDoH is rarely associated with financial rewards for primary care professionals,^24^ except for some innovative approaches.^26,27^ Fourth, there is little current evidence that clinicians can play effective roles in SDoH in primary care practice,^28,29^ particularly in small practice settings.^21^ In addition, comprehensive evidence-based recommendations about how to address SDoH have not yet been published.^30^

Given the complexity of this situation, primary care physicians sometimes lack the confidence to address social needs, and are afraid of contributing to poor health outcomes.^31–33^ Involvement in SDoH in the absence of solid and effective methods may raise the fear of unforeseen problems.^34^ Given these problems regarding SDoH in primary care settings, expecting primary care physicians to deal with SDoH may lead to further confusion and exhaustion, which may hinder the provision of high quality primary care to patients.

It remains unclear how primary care physicians feel about being required to take action on SDoH. To ensure that SDoH-related recommendations encourage primary care physicians to fulfil their role in relation to health inequity, the implications of the recommendations need to be clarified. The study aims to describe the perspectives of primary care physicians when asked to address SDoH.

## Method

### Setting

This qualitative study was conducted in Japan, and followed the Standards for Reporting Qualitative Research (SRQR).^35^ Recruitment, interviews, and discussion in the data analysis in this study were conducted online because of the COVID-19 pandemic.

In Japan, primary care is delivered both in the community and in hospital settings. Primary and secondary care are not always distinguished clearly.^36^ The distinction between family physicians, hospital family physicians, and hospitalists (engaging in both inpatient and outpatient care) is not clear in Japan, and they are sometimes collectively referred to as general medicine physicians.^37^ Most of them engage in primary care, and many subspecialists also play a role in primary care.^36^ The Japan Primary Care Association encourages each member to take action on daily practice including (i) prevention, (ii) education, (iii) research, (iv) partnership, and (v) advocacy, to eliminate unjust health inequities.^19^ As of 2022, this is the only recommendation published by official medical professional organisations in Japan.

### Reflexivity

The first author is a primary care physician and PhD student majoring in medical education. The first author is a member of the Japan Primary Care Association Commission on Social Determinants of Health, which published a recommendation about SDoH.^19^ The second author is a primary care physician and researcher in medical education. The third, fourth, and fifth authors are experts in medical education.

As a researcher standpoint, a social constructivism epistemology was adopted. Constructivists recognise that '*individuals construct different understandings based on their past experiences and knowledge'*.^38^ Social constructivism is a theory that learning is structured by the dynamic interaction between individuals and the environment, including other people, objects, and activities that occur there. This dynamism is seen in learners’ participation in the actual practice, especially when they are faced with conflicting ideas.^38,39^ The theory says that knowledge is a construction of the individual, and the learner participates in the learning process in an active way.^39^ The authors of the current study believe that participatory primary care physicians construct their own understandings of SDoH recommendations based on their experiences and clinical settings.

### Participants

Participants were recruited purposively to maintain diversity in years of experience, self-reported gender, and practice setting. Recruitment included direct request from researchers, notices in social network services, and recommendations from participants. All participants were primary care physicians, general medicine physicians, or residents in primary care. All participants were familiar with the concept of SDoH. Physicians that were involved in producing official recommendations regarding SDoH were excluded. Considering previous studies, an initial goal of recruiting 20 participants was set.^40,41^

### Data collection

To obtain multifaceted insights into participants’ ideas and feelings, love and breakup letter methodology was used.^42^ In this methodology, participants are asked to write love and breakup letters to an item or topic under discussion. These letters are used as triggers for subsequent interviews. The authors were concerned that opinions about being asked to address SDoH would be biased towards favourable responses because addressing SDoH is generally viewed as politically and ethically correct for primary care physicians. The love and breakup methodology has the potential to reveal both positive and negative feelings towards a topic.^43^ This methodology emerged from research on user experience,^42^ and was used to stimulate various thoughts and ideas that primary care physicians have when receiving recommendations about SDoH.

Data collection was performed according to the following four steps. First, participants voluntarily submitted written consent for research participation and completed a demographic data form. Second, participants read two recommendations^15,19^ that included recommendations for primary care physicians and family physicians to address SDoH in their daily practice. These two recommendations are the only SDoH-related recommendations published by the representative associations of primary care or family medicine, and were written in or officially translated into Japanese. Third, participants were given an explanation about the love and breakup letter method, and asked to write letters to a person who officially asks primary care physicians to address SDoH in their daily practice. To encourage participants to express their ideas and feelings freely, no further requirements were given about content, length, or wording. Some participants reported that it took about an hour or less to write these letters, and others reported that they ‘racked their brains’ for a few days. Fourth, participants were interviewed about their letters by the first author. The interviewer read these letters carefully before each interview, and asked participants about the meaning of their letters in detail and their feelings and thoughts in writing the letters. Table 1 shows examples of the love and breakup letters. The interviews were held from February 2022–May 2022. Each interview lasted approximately 15–20 minutes.

**Table 1.** Examples of love and breakup letters (not real letters that participants in this study wrote). SDoH = social determinants of health

**Love letter**
I am grateful to youYou always encourage me to pay attention to SDoHThanks to you, I can improve the quality of my practiceThanks to you, I can treat difficult patient encounters without having negative feelingsYou always enhance my identity as a primary care physicianTruly, there are many things that I cannot do on my ownHowever, you help me keep motivated to make our society betterThanks a lot
**Breakup letter**
Enough is enoughWhat you are saying is just fine-sounding talkIt is hard work to address patients' social difficulties, for which I receive little gratitudeWhatever you say, I cannot change societyYou are just exploiting my motivationYou impose enormous challenges on me in my busy practiceMy patience has reached its limitI don't even want to look at you anymore

### Data analysis

Every interview was recorded and transcribed verbatim. Anonymised transcripts and letters were analysed via thematic analysis using the framework approach.^44^ The analysis contained the following seven steps: verbatim transcription; familiarisation with the whole interview; initial coding; developing a working analytical framework; applying the framework to the whole data again; summarising data into the framework; and interpreting the data. Data analysis was conducted partly in parallel with data collection, and participant recruitment was completed after confirming that no additional theme emerged.^45^ The first and second authors coded the data, discussed it iteratively, and collapsed their analyses through the whole procedure. The other authors examined the analysis and revised the coding. All authors discussed the results iteratively and reached a consensus. Finally, all participants read the analysis and revised it if necessary.

## Results

A total of 21 participants were recruited, of which 38% were self-reported women. The median age was 40 years (range: 28–55 years) and the median duration of clinical experience was 10 years (range: 3–31 years). Table 2 shows demographic data.

**Table 2.** Participants’ demographics

Characteristic	
Median age, years (range)	40 (28–55)
Self-reported gender (% female)	38.1%
Duration of clinical experience, years	
3–6 (resident)	7
7–10	4
11–15	2
16–20	5
≥21	3
Main clinical setting, *n*	
Resident^a^	7
Clinic	4
Community hospital	6
Academic hospital	4

^a^In Japan, family medicine residents usually experience various clinical settings, including clinic, small-sized hospital, large hospital, and academic hospital.

The following four themes were identified from the qualitative analysis: (i) primary care physicians take pride in being expected to address SDoH; (ii) primary care physicians rely on the recommendations as a partner even in difficult situations; (iii) primary care physicians consider the recommendations to be bothersome, with unreasonable demands and challenges, especially when supportive surroundings are lacking; (iv) primary care physicians reconstruct the recommendations on the basis of their experience. Table 3 shows a summary of these themes and sub-themes.

**Table 3.** Summary of the themes and sub-themes

**Theme 1. Primary care physicians take pride in being expected to address SDoH**	
Sub-theme	Explanation
Integrability with primary care	Addressing SDoH is essential to primary care.Underlying factors are: Affinity with biopsychosocial model Best position (accessibility, comprehensiveness)
Excellence in primary care	Excellent primary care includes addressing SDoH. Experienced physicians: reaffirmation of the value in their practice Novice physicians: identity formation
**Theme 2. Primary care physicians rely on the recommendations as a partner even in difficult situations**	
Authoritative supporter	Recommendations validate: What primary care physicians have done Frustration and hesitation about patients’ social issues Background: addressing SDoH would be disregarded by other professionals
Encouraging friend	Busy practice leads physicians to ignorance of SDoH In supportive surroundings, they favour SDoH recommendation
**3. Primary care physicians consider the recommendations to be bothersome, with unreasonable challenges and demands, especially when supportive surroundings are lacking**	
Excessive burden	Recommendations impose on enormous challenges.Negative effects: Physicians disregard the importance of SDoH Physicians feel guilty Physicians underestimate their skills
Antipathy driven by unsupportive surroundings	Busy practice leads physicians to ignorance of SDoH In unsupportive surroundings, they disfavour SDoH recommendation
**4. Primary care physicians reconstruct the recommendations on the basis of their experience**	
Not following the recommendations literally	Recommendations are transformed and contextualised
Learning and practice based on real-world experience	Recommendations trigger multi-layered learning and practice

SDoH = social determinants of health.

### Primary care physicians take pride in being expected to address SDoH

Participants believed that they were in a unique position to address SDoH and they were proud to be relied on. They also recognised that addressing SDoH would enhance the quality of their practices.

#### Integrability with primary care

Participants reported that addressing SDoH was an essential component of primary care, and that it was a matter of course to be asked:

*'It* [addressing SDoH] *is not bothersome at all. It is my routine practice.'* (experience: 15 years; self-reported gender: female; setting: clinic; source: love letter)

Participants reported that addressing SDoH had an affinity with the biopsychosocial (BPS) model, and they could easily apply their skills:

*'BPS model is very closely related to care about SDoH.* […] [addressing SDoH] *is the very basis of our identity.'* (9 years; male; clinic; interview)

Participants considered primary care physicians as being in the best position to address SDoH because of their accessibility and comprehensiveness:

*'Primary care physicians have more opportunities to encounter patients with social and financial difficulties than subspecialists. Needless to say, we should be professional when seeing these patients.*' (3 years; male; resident; love letter)

#### Excellence in primary care

Participants believed that addressing SDoH allowed them to manage complex cases more robustly, and was thus a part of being an excellent primary care physician:

*'Thanks to your help, I can manage many issues. The trust that you have earned from patients, their families, and my staff are a valuable treasure.'* (15 years; female; clinic; love letter)

*'Understanding my patients more deeply via SDoH perspectives will help me mature as a physician.'* (4 years; male; resident; interview)

Experienced physicians perceived the recommendations as enhancing the value of their practice. Novices expressed admiration for the recommendations, and perceived them as being '*cool*':

*'What I have thought and experienced is verbalised in the recommendations. It is the very* […] *expression of what I really believe as a professional.'* (20 years; male; clinic; interview)

'*Keep being your cool self.*' (3 years; male; resident; love letter)

*'The recommendation has stimulated my feelings of honor and dignity. It is not appropriate for me to say this, as I am still inexperienced, but it* [addressing SDoH] *is what we have to do. We cannot avoid it.'* (3 years; male; resident; interview)

Some participants considered the recommendations as advocacy:

*'I sincerely admire you for appealing to society with such a wonderful statement.*' (20 years; male; clinic; love letter)

### Primary care physicians rely on the recommendations as a partner even in difficult situations

Participants favoured SDoH recommendations from the following two perspectives: authorities to validate their practices; and strongholds in times of hardship.

#### Authoritative supporter

Participants were grateful that the recommendations guaranteed the legitimacy of their commitment to SDoH in daily practice:

*'Whatever others say, you make me feel confident.'* (9 years; male; academic hospital; love letter)

*'I often wondered if addressing SDoH was just meddling. It* [the recommendation] *tells me that addressing SDoH is a meaningful initiative for patients and communities, in an evidence-based manner. Thus, I feel more confident.'* (15 years; male; community hospital; interview)

Participants also perceived that the recommendations verbalised and acknowledged the frustration and hesitation that they felt in their workplace:

*'I have experienced many cases where I felt powerless. I have the responsibility to deal with patients with difficult social challenges, but I often feel confused and frustrated.* […] *These recommendations empathise with my frustration and comfort me.'* (20 years; male; clinic; interview)

Underlying this gratefulness was the prediction that addressing SDoH would not be appreciated by other professionals:

*'The recommendation is an indulgence: it justifies our practice about SDoH. It recognises the value of what we have done.'* (29 years; male; community hospital; interview)

*'I was afraid that I might be seen as a hypocrite.* […] *I had a fear that other physicians might look at me strangely if I said loudly that addressing SDoH was important.'* (5 years; male; resident; interview)

The recommendations helped participants to communicate the importance and value of addressing SDoH with other staff:

*'You have helped me to speak with my colleagues to move beyond our differences of opinion.'* (3 years; male; resident; love letter)

#### Encouraging friend

The participants thought of the recommendations as a friend who pushed them to do the right thing regarding SDoH, even in the hardest of times:

'*There are many things I cannot do on my own regarding SDoH, but you keep me motivated.'* (5 years; male; resident; love letter)

*'When I am busy or distressed, I almost ignore SDoH. The recommendations remind me of what I value and encourage me to address SDoH.'* (15 years; male; community hospital; interview)

### Primary care physicians consider the recommendations to be bothersome, with unreasonable challenges and demands, especially when supportive surroundings are lacking

Participants disfavoured SDoH recommendations as a nuisance to impose on excessive burdens, especially in unsupportive practice surroundings. They reported the following three negative consequences: disregarding the importance of SDoH; feeling guilty; and underestimating their skills.

#### Excessive burden

Participants felt that the recommendations asked too much and that they would be overwhelmed by time-consuming and emotionally draining burdens. The wide-ranging scope of the recommendations contributed to the sense of being overwhelmed:

*'If I did everything you said, the work would never get done.'* (7 years; female; community hospital; breakup letter)

*'I have to see so many patients, I have limited time to spend, and I am so tired … I want to ignore you.'* (15 years; female; clinic; breakup letter)

This resulted in three negative consequences. First, some participants reported feeling a suspicion that addressing SDoH might not be worthwhile:

*'I cannot change anything. You are just exploiting our motivation with beautiful stories.'* (5 years; male; resident; breakup letter)

Second, some participants reported a sense of guilt in turning away from SDoH:

*'Please forgive me for not listening to you.'* (9 years; male; clinic; breakup letter)

Third, some participants reported that they negatively assessed their clinical competence to fully respond to the recommendations:

*'Other physicians may be doing well, but I’m not. I’m not that intelligent or clever.'* (20 years; male; community hospital; breakup letter)

*'You’re too good for me.'* (5 years; female; resident; breakup letter)

Participants were also concerned that an over-ambitious ideal would be counterproductive for spreading the philosophy of the recommendations:

*'I’m afraid that many people will turn their backs on the recommendations if they feel enforced to implement everything in them.'* (31 years; male; clinic; interview)

*'I don’t think there is much that physicians can actually do.'* (21 years; female; community hospital; interview)

#### Antipathy driven by unsupportive surroundings

Participants found the recommendations more bothersome when they confronted environments that were not suitable for the recommendations to be implemented:

*'The recommendations in the statement are difficult to implement in my current workplace, where there are few physicians and staff who are interested in SDoH.'* (10 years; female; academic hospital; interview)

'*I think that no one would praise me for addressing SDoH in the current situation.* […] *I know that working hard on SDoH won’t contribute to the hospital’s profits.*' (3 years; male; resident; interview)

In contrast, if participants worked in a cooperative environment and they had peers or colleagues to address SDoH with, they were more likely to perceive the recommendations positively, even in the midst of busy clinical practice:

*'It matters whether my colleagues are looking in the same direction and consider SDoH to be important. Without anyone else who acknowledges the importance of SDoH, I would feel very lonely, and I feel negative about these recommendations.* […] *On the contrary, with colleagues who share the same vision regarding SDoH, I would feel very positive, even if the statements recommend something I can’t do right now.'* (18 years; male; academic hospital; interview)

### Primary care physicians reconstruct the recommendations on the basis of their experience

Participants did not recognise that they should follow everything SDoH recommendations said. Rather, they considered SDoH recommendations as a trigger of multi-layered learning and practice.

#### Not following the recommendations literally

Participants recognised that reading the recommendations alone was not enough to change their practices. None of the participants reported that they followed the recommendations literally:

*'As for education and research, I have no idea what to do, because I have little experience in these domains.'* (4 years; male; resident; interview)

*'I could not gain insight into SDoH just by reading the recommendation.'* (4 years; female; resident; interview)

#### Learning and practice based on real-world experience

Participants reported that knowledge about SDoH and iterative reflection and practice was necessary to clearly understand what the recommendations said. They projected the recommendations into their clinical practice via various learning experiences:

*'I felt that SDoH had little to do with me, just after reading the recommendation. Through some educational sessions, I understood that I could and would address SDoH in my clinical practice. I finally realised that, by observing patients from multiple angles, I can recognise their hidden social determinants.'* (5 years; female; resident; interview)

*'It is difficult to just read the recommendation and then apply it to our clinical practice.* […] *Through iterative reflection and discussion with residents, I have got ideas about how to incorporate SDoH into our own practice.'* (18 years; female; academic hospital; interview)

Participants reported that realising the importance of SDoH in the real world was also important for an effective education:

*'Educational opportunities should be provided to increase physicians’ understanding that a lot of patients have socially complex backgrounds. Many physicians are still unaware that they do see such patients.'* (10 years; female; academic hospital; interview)

## Discussion

### Summary

The study explored primary care physicians’ views regarding recommendations that asked them to address SDoH in their clinical practices. The love and breakup letter methodology revealed ambiguous feelings and thoughts about the statements. Participants were proud of themselves as professionals to be asked to address SDoH and considered the recommendations to be helpful and supportive. Conversely, participants also thought of the recommendations as irritating and nagging, especially in the absence of peers with shared views regarding the importance of SDoH. Participants did not follow the recommendations literally, and they required reflective learning and practice to understand and educate themselves regarding SDoH in their clinical settings.

### Strengths and limitations

To the best of the authors' knowledge, this study is the first to examine how primary care physicians view recommendations about SDoH. These recommendations aim to reduce health inequity by changing the attitudes and behaviours of primary care professionals. The way in which such recommendations are received by primary care physicians is thus a matter of great concern. This study gathered negative as well as positive opinions. This methodology was not designed to dismiss recommendations or to cynically criticise efforts to address SDoH. Rather, the study revealed how the recommendations could be incorporated into education and practice regarding SDoH in clinical settings.

This study involved several limitations. Importantly, one participant reported severe distress when writing the breakup letter. The participant worked with a socially marginalised population and perceived writing a breakup letter as denying their own dedication. The participant could not fully express their ideas in the letter. Previous studies mentioned that some participants are uncomfortable and embarrassed to write and read their letters. In addition, researchers must be aware that the love and breakup letter methodology sometimes induces invasive emotional responses in participants. Instead of love and breakup letters, researchers can use 'fan' and 'admonition' letters, thereby maintaining the benefits of the methodology while avoiding unnecessary emotional disturbance.

In addition, the physicians who voluntarily participated in this research might have been those who had an interest in and a positive attitude towards SDoH. In particular, participatory residents, which represented one-third of all participants, might have a high affinity for SDoH because they were all under the Japan Primary Care Association family medicine expert training programme, which requires residents to address SDoH and submit a report. Thus, the findings might not reflect the opinions of primary care physicians who have little interest in SDoH or disagree with the commitment to addressing SDoH. However, this limitation was partially resolved by collecting negative views on the topic via the breakup letter.

A context of primary care in Japan should be mentioned to contextualise the findings. Although Japan has well-organised healthcare and social security systems, socioeconomic and health inequities still exist.^36,46^ In addition, physicians in Japan, especially residents, are chronically exposed to long working hours.^47^ Clinics and small-sized community hospitals in Japan are reimbursed under a fee-for-service model.^36^ This implies that most of SDoH-related clinical practice do not pay.

### Comparison with existing literature

Although many physicians believe in the importance of working to address patients’ social needs, few physicians are able to incorporate this approach into their practice.^32^ Primary care physicians recognised that the major disincentives to working on SDoH were a lack of time, staffing, and resources.^32,48,49^ These disincentives can promote commoditisation, commercialisation, and fragmentation of primary care, leading to inequalities in health care.^50^ In the current study, these difficulties were associated with negative opinions of the recommendations.

This study also indicated that, even if physicians felt burdened, supportive work conditions and cooperative team members were related to positive attitudes towards recommendations about SDoH. This relationship may have occurred because sharing tasks and responsibilities with members mitigated participants’ fears about lacking skills and resources, and helped them feel able to address SDoH in clinical settings.^26^ The ability to respond appropriately to patient social needs may thus reduce these mental stresses and improve self-efficacy.^51^ In addition, SDoH recommendations gave participants, especially younger ones, a sense of honour and dignity as primary care physicians. Primary care physicians tend to be unduly evaluated for their skills and roles.^52^ In Japan, there had been no official primary care training until recently,^53^ and unreasonable criticism from specialists may often reduce motivation to be a primary care physician.^54^ This context may partly explain why younger participants focus on their identity formation.

Being aware of unmet social needs in clinical practice might lead to further understanding of SDoH. Physicians can go beyond power inequalities between patients and physicians and bring patients’ social contexts into everyday encounters.^55,56^ Primary care physicians working in areas of lower socioeconomic conditions have more positive attitudes regarding their patients’ social problems.^48^ Physicians’ attitudes towards patients with social difficulties may be improved through changes in medical education,^57^ and reflective learning and practice about SDoH may play an important role in residents’ development.^58^

Participants did not literally implement the recommendations. Instead, they regarded the recommendations as encouraging and supportive, with positive implications for their clinical practice and further advancement of their existing efforts related to SDoH. The recommendations may not function as a norm to follow, but rather to support each physician to form their own clinical mindlines, or *'internalised and collectively reinforced tacit guidelines'*.^59,60^ Clinical mindlines are formed on the basis of various learning sources, reflection, and interactions with peers and colleagues, and this was also indicated in the current research.

### Implications for research and practice

Professional organisations that plan to develop and publish recommendations about SDoH should consider how their recommendations might be perceived by their target audience. By providing an opportunity to learn and discuss SDoH, their recommendations could help to change clinical practices more efficiently. For clinical supervisors, the current findings might provide useful tips about educating SDoH. Merely describing theoretical aspects of SDoH may not motivate trainees to change their attitudes and behaviours. Familiarising physicians with social determinants in a clinical setting and reflecting trainees’ experience may play a key role in postgraduate training. The COVID-19 pandemic has exacerbated health inequities.^61,62^ Primary care physicians potentially cope with the pandemic according to patients' social contexts.^63^ In the COVID-19 era, addressing SDoH in primary care should be promoted further.

Future research is needed to determine whether recommendations regarding SDoH and subsequent efforts of medical professionals can improve patient outcomes. In addition, future studies should elucidate the association between physicians’ working circumstances and attitudes towards such recommendations.

## Data

The dataset is not publicly available.
